# NCCBM, a Nomogram Prognostic Model in Breast Cancer Patients With Brain Metastasis

**DOI:** 10.3389/fonc.2021.642677

**Published:** 2021-04-29

**Authors:** Qiang Liu, Xiangyi Kong, Zhongzhao Wang, Xiangyu Wang, Wenxiang Zhang, Bolun Ai, Ran Gao, Yi Fang, Jing Wang

**Affiliations:** Department of Breast Surgical Oncology, National Cancer Center/National Clinical Research Center for Cancer/Cancer Hospital, Chinese Academy of Medical Sciences and Peking Union Medical College, Beijing, China

**Keywords:** brain metastasis, prognosis, breast cancer, nomogram, predictors

## Abstract

**Purpose:** Nomogram prognostic models could greatly facilitate risk stratification and treatment strategies for cancer patients. We developed and validated a new nomogram prognostic model, named NCCBM, for breast cancer patients with brain metastasis (BCBM) using a large BCBM cohort from the SEER (Surveillance, Epidemiology, and End Results) database.

**Patients and Methods:** Clinical data for 975 patients diagnosed from 2011 to 2014 were used to develop the nomogram prognostic model. The predictive accuracy and discriminative ability of the nomogram were determined by concordance index (C-index) and calibration curve. The results were validated using an independent cohort of 542 BCBM patients diagnosed from 2014 to 2015.

**Results:** The following variables were selected in the final prognostic model: age, race, surgery, radiation therapy, chemotherapy, laterality, grade, molecular subtype, and extracranial metastatic sites. The C-index for the model described here was 0.69 (95% CI, 0.67 to 0.71). The calibration curve for probability of survival showed good agreement between prediction by nomogram and actual observation. The model was validated in an independent validation cohort with a C-index of 0.70 (95% CI, 0.68 to 0.73).

**Conclusion:** We developed and validated a nomogram prognostic model for BCBM patients, and the proposed nomogram resulted in good performance.

## Introduction

Breast cancer is the most frequently diagnosed cancer in women worldwide and the second leading cause of cancer-related mortality in women in the United States (1). About 5 to 15% of women with breast cancer were diagnosed with central nervous system (CNS) metastasis; however, the incidence of breast cancer patients with brain metastasis (BCBM) was reported to be as high as 30% (2). The development of brain metastasis in breast cancer patients results in a significant reduction in overall survival duration (3). The median survival time for all subtypes of patients with breast cancer with untreated brain metastasis is only 10 months and varies with different clinical parameters (3). Prognostic models that accurately predict the survival of BCBM in the modern era of breast cancer treatments are essential to optimize the management of BCBM.

The prognosis of BCBM varies largely with different clinical features; therefore, prognostic models are warranted to aid the clinical decision and possibly help in stratifying patients for further therapy. In the past few decades, a few prognostic models has been developed to predict the prognosis for BCBM; however, these models showed limited performance when applied to external validation cohorts, thereby remaining insufficient, and the routine use of these prognostic models is challenged (4). The first prognostic model for BCBM was developed in 1997 by the Radiation Therapy Oncology Group (RTOG) using a recursive partitioning analysis (RPA) method (4) and was replaced by the prognostic assessment (GPA) model 11 years later (5). In 2010, the GPA methodology was adapted to construct diagnosis-specific GPA classes (DS-GPA) to predict survival in patients with brain metastasis from breast cancers and other tumors (6). It is worthy to note that evidence showed clear separation between subgroups of patients with breast cancer and brain metastases (7). In 2012, Weil et al. developed a prognostic nomogram for BCBM with a concordance index (C-index) of 0.67 in a population of 261 women, comparing the performance of the nomogram with aforementioned prognostic models; Kattan et al. developed a nomogram based on de-identified data for 2,367 patients with brain metastasis from seven RTOG randomized trials (8). Paul W Sperduto et al. developed a model named Breast GPA with a larger contemporary cohort; they found the median survival has improved modestly but varies widely by diagnosis-specific prognostic factors (9).

In the present study, we developed and validated a nomogram prognostic model in a population of 1,517 patients. We investigated the sociodemographic and clinicopathologic predictors associated with BCBM and constructed a robust nomogram for predicting BCBM survival at 6 months, 1 year, and 2 years. The proposed nomogram was validated in an independent external validation cohort and showed good performance.

## Methods

### Study Population and Design

Since the Surveillance, Epidemiology, and End Results (SEER) database began collecting information on the molecular subtypes and sites of distant metastasis in 2010, BCBM cases at the time of initial cancer diagnosis from 2010 to 2015 were enrolled in the present study. The inclusion criteria were as follows: (1) Presence of brain metastasis; (2) clear follow-up information; (3) reporting source was neither autopsy nor death certificate only. The exclusion criteria were as follows: (1) tumors of uncertain origin and (2) cases with duplicated record. A total of 975 cases that were diagnosed from 2010 to 2013 were assigned to the training cohort and used to develop the nomogram prognostic model. The 542 cases diagnosed from 2014 to 2015 were assigned to the independent validation cohort and used to validate the model. This study was approved by the institutional review board at the Ethics Committee of Cancer Hospital of Chinese Academy of Medical Sciences, and written informed consent was waived since data were derived from the SEER database.

### Variable Selection

Overall survival (OS) was defined as the length of time from diagnosis to death or last contact and used as the primary outcome. The following variable data were extracted and classified according to the codes in the SEER database: sex, age, race, marital status at diagnosis, insurance recode (10), breast tumor laterality, tumor primary site, molecular subtype, histological grade, pathological pattern [infiltrating duct carcinoma (IDC), lobular carcinoma (LC), infiltrating ductal and lobular carcinoma (IDLC), cribriform carcinoma, tubular adenocarcinoma, mucinous adenocarcinoma, infiltrating duct mixed with other types of carcinoma (IDM), ductal carcinoma, micropapillary, and others], American Joint Committee on Cancer (AJCC) T stage, AJCC N stage, surgery recode, radiation recode, chemotherapy recode, survival in months, and number of extracranial metastatic sites.

### Statistical Analysis

A nomogram was constructed based on the results of multivariate analysis and by using the rms package (11) of in R version 3.6.3 (http://www.r-project.org/). A final model selection was performed by a backward stepdown selection process with the Akaike information criterion (12). The performance of the nomogram was assessed by C-index and measured by comparing nomogram-predicted vs. observed Kaplan–Meier estimates of survival probability. Bootstraps with 1,000 resamples were used for these activities. C-index and 95% confidence interval (CI) were computed using *survcomp* package (13) in R. The calibration plots were generated by comparing the nomogram-predicted probability of OS at 6 months, 1 year, and 2 years with the observed survival probability. The interpretation of this index is similar to that of a receiver–operator curve: an index of 1.0 indicates a model that is perfectly concordant with the dataset; an index of 0.0 suggests perfect discordance (14). *P* < 0.05 was considered statistically significant.

## Results

### Characteristics of the Training and Validation Cohorts

In total, 1,517 cases that did not contain any missing variables were included in this study. Based on year of diagnosis, the included cases were divided into two distinct groups: cases that were diagnosed from 2010 to 2013 (*n* = 975) were used as the training cohort, whereas cases that were diagnosed from 2014 to 2015 (*n* = 542) were used as the validation cohort. The median follow-up time was 5 years (95% CI, 4.5–5.33 years) for the training cohort and 1.83 years (95% CI, 1.67–2 years) for the validation cohort. Characteristics of the two datasets are summarized in Table 1.

**Table 1:** Demographics and clinicopathologic characteristics of breast cancer patients with brain metastasis.

**Variables**	**Training cohort (*n* = 975)**	**Validation cohort (*n* = 542)**	**Overall (*n* = 1,517)**
	**No. of patients (%)**	**No. of patients (%)**	**No. of patients (%)**
**Age (years)**			
<40	53 (5.4%)	35 (6.5%)	88 (5.8%)
40–49	142 (14.6%)	64 (11.8%)	206 (13.6%)
50–59	271 (27.8%)	162 (29.9%)	433 (28.5%)
60–69	279 (28.6%)	163 (30.1%)	442 (29.1%)
70–79	161 (16.5%)	75 (13.8%)	236 (15.6%)
≥80	69 (7.1%)	43 (7.9%)	112 (7.4%)
**Sex**			
Male	11 (1.1%)	9 (1.7%)	20 (1.3%)
Female	964 (98.9%)	533 (98.3%)	1497 (98.7%)
**Race**			
White	613 (62.9%)	319 (58.9%)	932 (61.4%)
Black	182 (18.7%)	101 (18.6%)	283 (18.7%)
Hispanic	123 (12.6%)	65 (12.0%)	188 (12.4%)
Asian/Pacific Islander	53 (5.4%)	51 (9.4%)	104 (6.9%)
Other	4 (0.4%)	6 (1.1%)	10 (0.7%)
**Marital status**			
None-single	701 (71.9%)	371 (68.5%)	1072 (70.7%)
Single	224 (23.0%)	133 (24.5%)	357 (23.5%)
Unknown	50 (5.1%)	38 (7.0%)	88 (5.8%)
**Insurance**			
Uninsured	69 (7.1%)	26 (4.8%)	95 (6.3%)
Insured	884 (90.7%)	500 (92.3%)	1384 (91.2%)
Unknown	22 (2.3%)	16 (3.0%)	38 (2.5%)
**Laterality**			
Left	464 (47.6%)	250 (46.1%)	714 (47.1%)
Right	434 (44.5%)	250 (46.1%)	684 (45.1%)
Bilateral	70 (7.2%)	37 (6.8%)	107 (7.1%)
Unknown	7 (0.7%)	5 (0.9%)	12 (0.8%)
**Primary site**			
Upper-outer	185 (19.0%)	108 (19.9%)	293 (19.3%)
Upper-inner	37 (3.8%)	28 (5.2%)	65 (4.3%)
Lower-inner	26 (2.7%)	16 (3.0%)	42 (2.8%)
Lower-outer	41 (4.2%)	20 (3.7%)	61 (4.0%)
Overlapping	159 (16.3%)	90 (16.6%)	249 (16.4%)
Central	39 (4.0%)	26 (4.8%)	65 (4.3%)
Breast_NOS	466 (47.8%)	246 (45.4%)	712 (46.9%)
Other	22 (2.3%)	8 (1.5%)	30 (2.0%)
**Surgery**			
Surgery not performed	810 (83.1%)	479 (88.4%)	1289 (85.0%)
Surgery performed	158 (16.2%)	58 (10.7%)	216 (14.2%)
Unknown	7 (0.7%)	5 (0.9%)	12 (0.8%)
**Radiation**			
Radiotherapy not performed	16 (1.6%)	5 (0.9%)	21 (1.4%)
Radiotherapy performed	596 (61.1%)	321 (59.2%)	917 (60.4%)
None/Unknown	363 (37.2%)	216 (39.9%)	579 (38.2%)
**Chemotherapy**			
No/Unknown	464 (47.6%)	263 (48.5%)	727 (47.9%)
Yes	511 (52.4%)	279 (51.5%)	790 (52.1%)
**Histology**			
IDC	606 (62.2%)	339 (62.5%)	945 (62.3%)
LC	50 (5.1%)	23 (4.2%)	73 (4.8%)
IDLC	23 (2.4%)	15 (2.8%)	38 (2.5%)
IDM	11 (1.1%)	7 (1.3%)	18 (1.2%)
Mucinous	6 (0.6%)	3 (0.6%)	9 (0.6%)
Tubular	0 (0%)	0 (0%)	0 (0%)
DCM	1 (0.1%)	2 (0.4%)	3 (0.2%)
Other	278 (28.5%)	153 (28.2%)	431 (28.4%)
**AJCC T**			
T1	106 (10.9%)	67 (12.4%)	173 (11.4%)
T2	198 (20.3%)	109 (20.1%)	307 (20.2%)
T3	104 (10.7%)	78 (14.4%)	182 (12.0%)
T4	322 (33.0%)	158 (29.2%)	480 (31.6%)
TX	216 (22.2%)	116 (21.4%)	332 (21.9%)
T0	29 (3.0%)	14 (2.6%)	43 (2.8%)
**AJCC N**			
N0	241 (24.7%)	137 (25.3%)	378 (24.9%)
N1	368 (37.7%)	223 (41.1%)	591 (39.0%)
N2	93 (9.5%)	43 (7.9%)	136 (9.0%)
N3	127 (13.0%)	58 (10.7%)	185 (12.2%)
NX	146 (15.0%)	81 (14.9%)	227 (15.0%)
**Grade**			
Grade I	31 (3.2%)	18 (3.3%)	49 (3.2%)
Grade II	264 (27.1%)	126 (23.2%)	390 (25.7%)
Grade III	365 (37.4%)	220 (40.6%)	585 (38.6%)
Grade IV	13 (1.3%)	2 (0.4%)	15 (1.0%)
Unknown	302 (31.0%)	176 (32.5%)	478 (31.5%)
**Subtype**			
HR+/HER2-	359 (36.8%)	204 (37.6%)	563 (37.1%)
HR+/HER2+	142 (14.6%)	80 (14.8%)	222 (14.6%)
HR-/HER2+	108 (11.1%)	67 (12.4%)	175 (11.5%)
HR-/HER2-	172 (17.6%)	102 (18.8%)	274 (18.1%)
Unknown	194 (19.9%)	89 (16.4%)	283 (18.7%)
**Extracranial metastatic sites**			
No	184 (18.9%)	104 (19.2%)	288 (19.0%)
One	370 (37.9%)	186 (34.3%)	556 (36.7%)
Two	270 (27.7%)	159 (29.3%)	429 (28.3%)
Three	142 (14.6%)	91 (16.8%)	233 (15.4%)
Unknown	9 (0.9%)	2 (0.4%)	11 (0.7%)

### Nomogram Prognostic Model in Training Cohort

**Figure 1:**
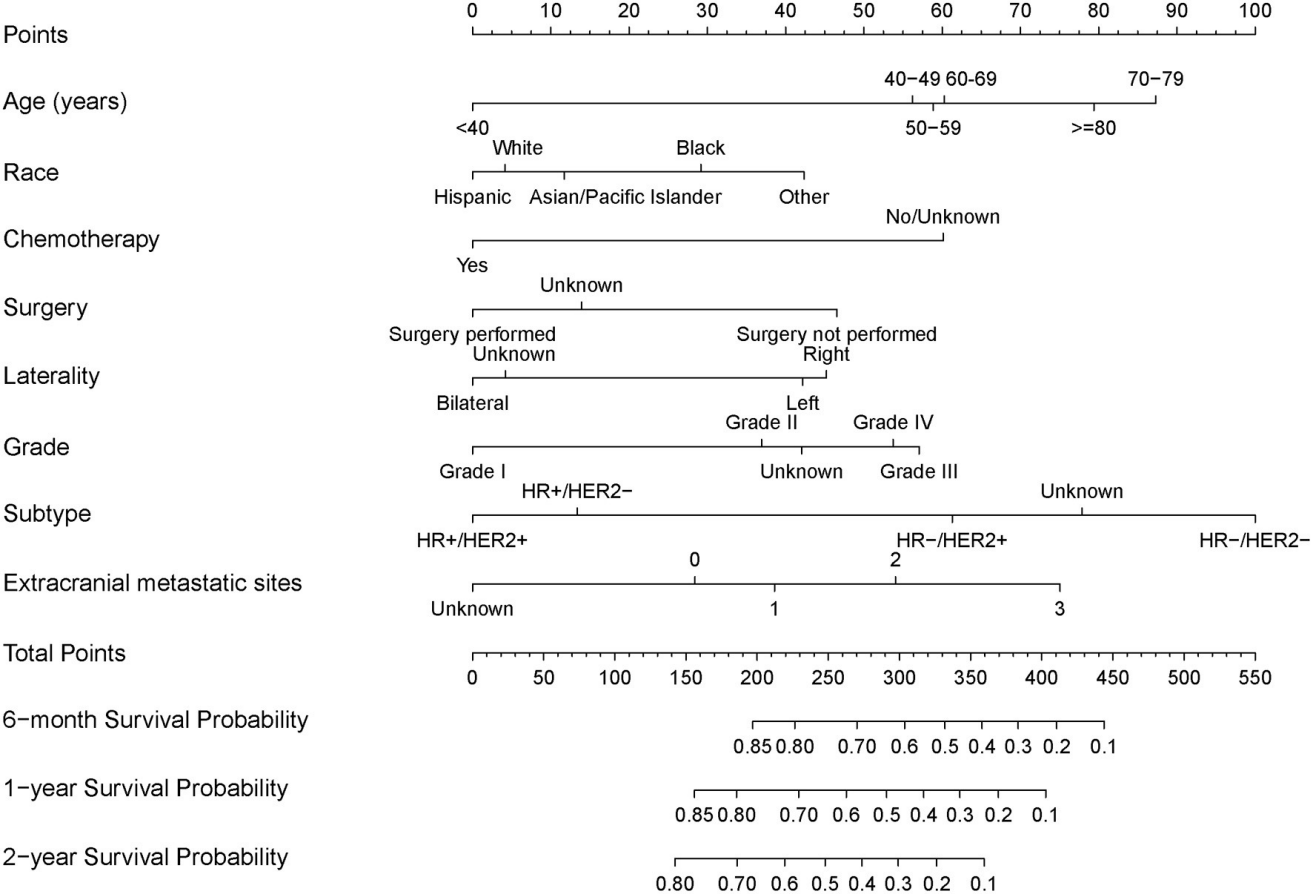
Nomograms for predicting 6-month, 1-year, and 2-year overall survival (OS) of breast cancer patients with brain metastasis.

**Figure 2:**
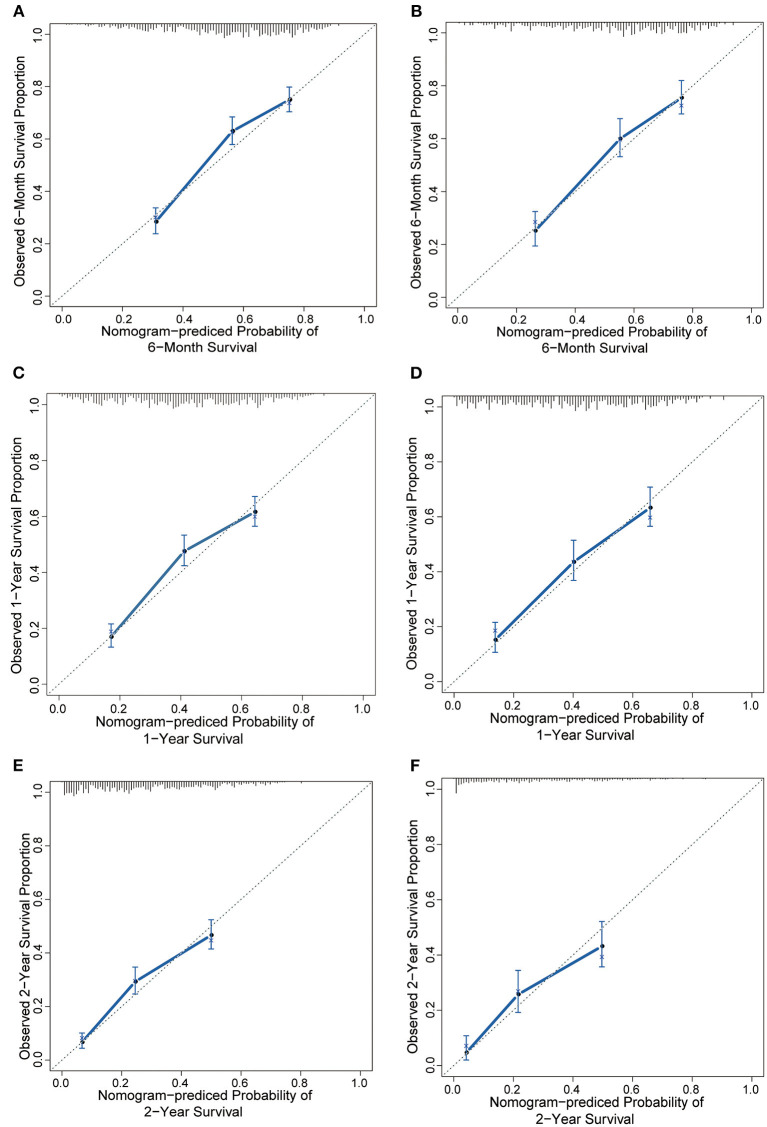
The calibration curve for predicting patient survival at **(A)** 6 months, **(C)** 1 year, and **(E)** 2 years in the training cohort and at **(B,D,F)** 6 months, 1 year, and 2 years in the validation cohort. Nomogram-predicted probability of overall survival is plotted on the x-axis; actual overall survival is plotted on the y-axis.

The results of the univariate analysis are listed in Supplementary Table 1. Multivariate analyses demonstrated that age, race, surgery, radiation therapy, chemotherapy, laterality, grade, molecular subtype, and extracranial metastatic sites were independent risk factors for OS (Table 2). The prognostic nomogram that integrated all significant independent factors for OS in the primary cohort is shown in Figure 1. The C-index for OS prediction was 0.69 (95% CI, 0.67 to 0.71). The calibration plot for the probability of survival at 6 months, 1 year, and 2 years showed a good agreement between the prediction by nomogram and actual observation (Figures 2A,C,E).

**Table 2:** Multivariate analysis of the training cohort.

**Variable**	**HR**	**95%CI**	***P*-value**
**Age (years)**			
<40	1	[Reference]	
40–49	1.85	1.27–2.71	0.001
50–59	1.87	1.31–2.67	0.001
60–69	1.95	1.36–2.79	*P* < 0.001
70–79	2.68	1.83–3.91	*P* < 0.001
≥80	2.34	1.5–3.63	*P* < 0.001
**Race**			
White	1	[Reference]	
Black	1.22	1.01–1.47	0.039
Hispanic	0.95	0.76–1.19	0.676
Asian/Pacific Islander	1.11	0.8–1.53	0.533
Other	1.99	0.61–6.44	0.251
**Laterality**			
Left	1	[Reference]	
Right	1.02	0.88–1.18	0.797
Bilateral	0.65	0.47–0.91	0.011
Unknown	0.68	0.29–1.58	0.375
**Surgery**			
Surgery not performed	1	[Reference]	
Surgery performed	0.6	0.49–0.74	*P* < 0.001
Unknown	0.77	0.31–1.9	0.565
**Chemotherapy**			
No/Unknown	1	[Reference]	
Yes	0.52	0.44–0.61	*P* < 0.001
**Grade**			
Grade I	1	[Reference]	
Grade II	1.64	1.04–2.58	0.034
Grade III	2.02	1.28–3.19	0.003
Grade IV	1.97	0.93–4.19	0.077
Unknown	1.74	1.09–2.79	0.02
**Subtype**			
HR+/HER2-	1	[Reference]	
HR+/HER2+	0.86	0.68–1.1	0.228
HR-/HER2+	1.69	1.3–2.18	*P* < 0.001
HR-/HER2-	2.54	2.02–3.19	*P* < 0.001
Unknown	1.87	1.5–2.33	*P* < 0.001
**Extracranial metastatic sites**			
No	1	[Reference]	
One	1.12	0.92–1.38	0.259
Two	1.27	1.01–1.59	0.039
Three	1.6	1.24–2.05	*P* < 0.001
Unknown	0.62	0.29–1.33	0.217

### External Validation of the Nomogram

In the validation cohort, we test the nomogram prognostic model using the same model parameters as the developed nomogram in the training cohort. Our results indicated the C-index of the nomogram for predicting OS was 0.70 (95% CI, 0.68 to 0.73), and a calibration curve also showed excellent agreement between prediction and observation in the probability of 6 months, 1 year, and 2 years (Figures 2B,D,F). These results suggested that predictions in an independent data set were excellent and therefore confirmed the exportability of the model.

## Discussion

In this study, the NCCBM prognostic model was developed and validated using a large cohort of BCBM cases across the United States. This NCCBM nomogram, based on routinely available demographic, staging, and treatment information, can predict the survival probability for individual BCBM, which might be helpful for assisting clinicians in making therapy decisions.

### Prognostic Predictors for BCBM

A plethora of previous studies have reported the prognostic factors for survival among BCBM, including tumor subtype, age, Karnofsky Performance Status, number of brain metastases, systemic chemotherapy, surgical resection, interval from first cancer diagnosis to brain metastases, size of primary tumor, presence/degree of extracranial metastases, primary tumor control, dose of radiation, and solitary metastases. In the present study, we found the prognostic variables for BCBM were as follows: age, race, surgery, radiation therapy, chemotherapy, laterality, grade, molecular subtype, and extracranial metastatic sites. Some variables we reported were consistent with previous results including tumor subtype, age, treatment information (surgery, chemotherapy, and radiation), and extracranial metastases. We also found the race and tumor grade were independent predictors for survival of BCBM (Table 2). We noticed a series of interesting results. Firstly, patients with Grade III represent the worst prognosis [hormone receptor (HR): 2.02; 95% CI, 1.28–3.19; *p* < 0.003] when compared with Grade I, but not Grade IV. Secondly, older patients indicated worse outcome generally, but 70- to 79-year-old patients showed the worst outcome (HR: 2.68; 95% CI, 1.83–3.91; *p* < 0.001), although not patients older than 80 years.

### Nomogram Prognostic Model for BCBM

The NCCBM nomogram described in this study was developed based on the SEER database, encompassing approximately 28% of the US population, which is a significant strength for future clinical application compared with using limited single institutional data. The performance of the NCCBM nomogram was assessed by calibration and discrimination. Calibration is defined as the ability to estimate the agreement between the nomogram estimated survival and the observed survival. In the present study, the calibration plots showed excellent agreement in both the training and validation cohorts, which suggested the reliability of the NCCBM nomogram. Discrimination is defined as the ability to distinguish between patients who experience an event and those who do not experience it. The discrimination of the NCCBM nomogram was assessed by the C-index. The C-index of the NCCBM was 0.69 (95% CI, 0.67 to 0.71) in the training cohort and 0.70 (95% CI, 0.68 to 0.73) in the validation cohort, suggesting the robust performance of this prognostic model.

As was reported by Marko et al. in 2012 (15), their nomogram based on a population of 261 women showed a C-index of 0.67 with only internal validation, and when compared with RPA, GPA, original DS-GPA and modified DS-GPA models. Although in a more representative population cohort, the NCCBM nomogram showed a better performance than the aforementioned prognostic models. More recently, Song et al. reported a novel nomogram for predicting OS for BCBM with a C-index of 0.735 (16); however, this nomogram was developed only based on a limited patient size from a single institution, which is not a good representation of population, and the performance has not been validated in an external cohort. In summary, the NCCBM nomogram represents a wide population and showed a moderate predictive effect on prognosis of BCBM.

### Potential Limitations

Despite the promising findings of the present study, this study should be considered in the context of its limitations. Firstly, although the SEER database represents about 30% of the US population, clinical data on tumor subtype and distant metastatic sites was collected only after 2010 in the SEER database and therefore limited the sample size of this study. Secondly, information about disease recurrence or subsequent sites of disease involvement was not collected in the SEER database (17); hence, we were unable to investigate patients who developed brain metastases later in their disease course. Thus, there might be some patients who subsequently developed brain metastases later in the disease course who would not be included in our analysis, which may lead to bias of the results. Future investigations using alternative data sources should be carried out to address this important point. Thirdly, detailed treatment information for patients with brain metastases is not recorded in the SEER database; thus, we cannot comment on more on this. Fourth, since information relating to Karnofsky Performance Status was not available in the SEER, we were unable to compare the prediction effect of NCCBM nomogram and other prognostic models directly. In addition, when applying it to other countries and areas, external validation should be conducted to test its validity. In summary, further prospective study using more detailed clinical data should be carried out to validate the robustness of this model before clinical application and extension.

## Conclusions

The current study used a Cox proportional hazards regression in conjunction with a nomogram representation to construct a robust predictive model of survival of breast cancer patients with bone metastasis. The NCCBM model is based on a combination of eight clinical and molecular features that should be readily available to clinicians treating patients with breast cancer, and our validation results suggest that this model should be highly reproducible in similar patient populations.

## Data Availability Statement

The original contributions presented in the study are included in the article/Supplementary Material, further inquiries can be directed to the corresponding author/s.

## Ethics Statement

The studies involving human participants were reviewed and approved by the institutional review board at Ethics Committee of Cancer Hospital of Chinese Academy of Medical Sciences. Written informed consent was waived as the data were derived from SEER database.

## Author Contributions

JW, YF, and QL: conception and design. QL: development of methodology. XK: acquisition of data. QL, XK, and ZW: analysis and interpretation of data. QL, XK, and RG: writing, review, and/or revision of the manuscript. XW, BA, and XK: administrative, technical, or material support. JW and YF: study supervision. All authors read and approved the final manuscript.

## Conflict of Interest

The authors declare that the research was conducted in the absence of any commercial or financial relationships that could be construed as a potential conflict of interest.

## Abbreviations

SEER: surveillance, epidemiology, and end results
BCBM: breast cancer patients with brain metastasis
OR: odds ratio
HR: hormone receptor
HER2: human epidermal growth factor receptor 2
LC: lobular carcinoma
IDC: infiltrating duct carcinoma.
