# Supplementary material for: NCCBM, a Nomogram Prognostic Model in Breast Cancer Patients With Brain Metastasis
Source: Front Oncol. 2021 Apr 29;11:642677. doi: 10.3389/fonc.2021.642677 (PMC8116746; doi:10.3389/fonc.2021.642677)
Supplement: Supplementary file 1 [file Table_1.docx]

| **Table S1. Univariate Analysis of the Training Cohort** | | | |
| --- | --- | --- | --- |
| **Variables** | **HR** | **95%CI** | **pvalue** |
| **Age (years)** |  |  |  |
| <40 | 1 | [Reference] |  |
| 40-49 | 1.18 | 0.89-1.58 | 0.255 |
| 50-59 | 1.28 | 0.98-1.68 | 0.066 |
| 60-69 | 1.43 | 1.1-1.87 | 0.008 |
| 70-79 | 2.01 | 1.52-2.65 | 0 |
| >=80 | 2.43 | 1.78-3.32 | 0 |
| **Sex** |  |  |  |
| Male | 1 | [Reference] |  |
| Female | 0.9 | 0.54-1.49 | 0.67 |
| **Race** |  |  |  |
| White | 1 | [Reference] |  |
| Black | 1.26 | 1.09-1.46 | 0.001 |
| Hispanic | 0.84 | 0.7-1 | 0.053 |
| Asian/Pacific Islander | 0.96 | 0.76-1.21 | 0.703 |
| Other | 0.77 | 0.36-1.61 | 0.484 |
| **Laterality** |  |  |  |
| Left | 1 | [Reference] |  |
| Right | 1.03 | 0.91-1.15 | 0.672 |
| Bilateral | 1.04 | 0.83-1.31 | 0.703 |
| Unknown | 0.85 | 0.46-1.59 | 0.616 |
| **Surgery** |  |  |  |
| Surgery not performed | 1 | [Reference] |  |
| SurgerySurgery performed | 0.61 | 0.52-0.72 | 0 |
| SurgeryUnknown | 0.42 | 0.19-0.94 | 0.035 |
| **Radiation** |  |  |  |
| Radiotherapy not performed | 1 | [Reference] |  |
| Radiotherapy performed | 0.64 | 0.41-1.01 | 0.058 |
| None/Unknown | 0.93 | 0.59-1.47 | 0.761 |
| **Chemotherapy** | |  |  |
| None/Unknown | 1 | [Reference] |  |
| ChemotherapyYes | 0.54 | 0.48-0.6 | 0 |
| **Histology** |  |  |  |
| IDC | 1 | [Reference] |  |
| LC | 0.94 | 0.72-1.24 | 0.675 |
| IDLC | 0.76 | 0.53-1.1 | 0.151 |
| IDM | 1.49 | 0.91-2.45 | 0.113 |
| Mucinous | 0.74 | 0.35-1.55 | 0.419 |
| DCM | 1.59 | 0.51-4.96 | 0.42 |
| Other | 1.23 | 1.09-1.4 | 0.001 |
| **AJCC T** |  |  |  |
| T1 | 1 | [Reference] |  |
| T2 | 0.88 | 0.72-1.09 | 0.244 |
| T3 | 0.94 | 0.75-1.18 | 0.609 |
| T4 | 1.05 | 0.86-1.26 | 0.646 |
| TX | 1.06 | 0.87-1.3 | 0.564 |
| T0 | 0.72 | 0.49-1.05 | 0.087 |
| **AJCC N** |  |  |  |
| N0 | 1 | [Reference] |  |
| N1 | 1.01 | 0.88-1.17 | 0.885 |
| N2 | 0.76 | 0.61-0.95 | 0.017 |
| N3 | 1.02 | 0.84-1.23 | 0.877 |
| NX | 1.17 | 0.98-1.41 | 0.086 |
| **Grade** |  |  |  |
| I | 1 | [Reference] |  |
| II | 1.26 | 0.89-1.79 | 0.192 |
| III | 1.56 | 1.11-2.2 | 0.011 |
| IV | 1.24 | 0.64-2.38 | 0.526 |
| Unknown | 1.58 | 1.11-2.23 | 0.01 |
| **Subtype** |  |  |  |
| HR+/HER2- | 1 | [Reference] |  |
| HR+/HER2+ | 0.73 | 0.61-0.88 | 0.001 |
| HR-/HER2+ | 1.07 | 0.88-1.29 | 0.517 |
| HR-/HER2- | 1.92 | 1.65-2.25 | 0 |
| Unknown | 1.8 | 1.54-2.1 | 0 |
| **Extracranial metastatic sites** | | |  |
| No | 1 | [Reference] |  |
| One | 1.01 | 0.86-1.18 | 0.906 |
| Two | 1.08 | 0.91-1.28 | 0.372 |
| Three | 1.41 | 1.17-1.7 | 0 |
| Unknown | 1.39 | 0.76-2.55 | 0.286 |
| Abbreviations: HER2, human epidermal growth factor receptor 2; HR, hormone receptor; NOS, not otherwise specified; IDC, infiltrating duct carcinoma; LC, lobular carcinoma; IDLC, infiltrating duct and lobular carcinoma; IDM, infiltrating duct mixed with other types of carcinoma; Mucinous, mucinous adenocarcinoma; Tubular, tubular adenocarcinoma; DCM, ductal carcinoma, micropapillary; + denotes positive; − denotes negative. | | | |
